# Challenging role of Wnt5a and its signaling pathway in cancer metastasis (Review)

**DOI:** 10.3892/etm.2014.1676

**Published:** 2014-04-11

**Authors:** NENG ZHU, LI QIN, ZHIGANG LUO, QIONG GUO, LUOYAN YANG, DUANFANG LIAO

**Affiliations:** 1 Department of Urology, Second Xiangya Hospital, Central South University, Changsha, Hunan 410011, P.R. China; 2 Department of Urology, Second Affiliated Hospital of South China University, Hengyang, Hunan 421001, P.R. China; 3 Institute of Pharmacy and Pharmacology, University of South China, Hengyang, Hunan 421001, P.R. China; 4 School of Pharmacy, Hunan University of Chinese Medicine, Changsha, Hunan 410208, P.R. China

**Keywords:** Wnt5a, cancer, metastasis, signaling pathway

## Abstract

Wnt5a is a noncanonical signaling member of the wingless-related/mouse mammary tumor virus integration family, which is involved in a wide range of cellular processes, particularly in cancer development and metastasis. Accumulating evidence indicates that Wnt5a exhibits paradoxical effects in various types of cancer metastasis. Therefore, the Wnt5a signaling cascade in cancer metastasis appears to be complex and may depend on binding receptors, downstream effectors, exogenous inhibitors and tumor microenvironments, as well as the extracellular matrix, particularly cell/tissue-tropic contexts. The aim of the present study was to summarize the previous findings on the roles of Wnt5a and the potential mechanisms in various types of cancer metastasis. Furthermore, it is reasonable to hypothesize that Wnt5a and the involved signaling pathways may become molecular targets in the treatment of cancer metastasis.

## 1. Introduction

Metastasis is the most important biological characteristic of malignant tumors and is currently the main cause of cancer-associated mortality. Cancer invasion and metastasis are two essential steps in the complex tumor development process and result from a series of biological changes in cancer and stromal cells (1). Firstly, cancer cells acquire the movement characteristics of epithelial mesenchymal cells, which is accompanied by a decrease in intercellular adhesion. During this period, the extracellular matrix adhesion abilities of cancer cells are decreased. Therefore, cancer cells depart from the primary cancer tumor and are transported through membranes easily. The cancer cells then enter the vascular or lymphatic vessels. Finally, the new proliferated high-colony cells are established in the distant sites (2). In previous years, studies have found that the Wnt signaling pathway is closely associated with other signaling cellular elements, including adhesion molecules, proteases and angiogenic factors, during the cancer metastasis process (3,4).

## 2. Wnt5A is involved in noncanonical Wnt signaling

WNT genes encode a family of 38–43 kDa glycoproteins, which are growth factors typically secreted by cells. Wnt proteins were first identified in mammals as the proto-oncogenic integration site for the mouse mammary tumor virus. These proteins include a hydrophobic signal peptide, highly conserved cysteine residues and N-linked glycosylation sites, with the absence of additional transmembrane domains (5). There are common characteristics in the primary structure of the Wnt family proteins, including a signal sequence for secretion, glycosylation sites essential for activity and a cysteine-rich domain that is involved in protein folding. These proteins associate with cell surfaces and the extracellular matrix, and a number are known to bind to the Frizzled family of receptors (Fzd) that resemble typical G protein-coupled receptors (6). To date, at least 19 Wnt members have been shown to be present in human and mice, with functions contributing to the regulation of a wide range of cellular processes, particularly in development. Wnt proteins have also been implicated in tumor formation. The WNT5A gene was first identified in mice, with multiple transcripts encoding a cysteine-rich protein consisting of 379 amino acids, whose temporal expression correlated with the spatial patterning and morphogenesis (7). Clark *et al* (8) reported the cloning and sequencing of several overlapping cDNA molecules that encoded the 4.1 kb human homologue of Wnt5a. The mature human Wnt5a protein contains 343 residues and has >93% homology to the reported sequences of other Wnt5a proteins (>99% homologous to mouse Wnt5a). Using a combination of Southern blotting, polymerase chain reaction amplification and *in situ* hybridization, the human WNT5A gene was mapped to chromosome 3p14-p21 (9).

Wnt proteins activate a number of signaling pathways, which can be divided into two general categories; the canonical β-catenin pathway and the noncanonical β-catenin independent pathways. In the canonical pathway, a Wnt protein (such as Wnt1, Wnt3a and Wnt7a) binds to the receptors and triggers a cascade that is mediated by dishevelled proteins, inhibiting glycogen synthase kinase-3β (GSK-3β) activity. Inactivation of GSK-3β results in the hypophosphorylation of β-catenin, which escaped from the complex that formed with adenomatous polyposis coli (APC) and Axin, and degraded by the ubiquitin/proteasome system. The accumulated free β-catenin enters the nucleus, activates the Tcf/Lef transcription factors and in turn triggers the transcription of a set of target genes, ultimately leading to the regulation of cell proliferation and cell apoptosis, as well as cell transformation (10,11).

Wnt5a is a representative ligand that activates the noncanonical Wnt signaling pathways, which are defined as being independent from the β-catenin pathway. Noncanonical Wnt cascades are diverse and in a number of cases, hard to define. The pathways are classified into the following categories for clarity and simplicity: i) Wnt/planar cell polarity signaling; ii) Wnt-cyclic guanosine monophosphate/Ca^2+^ signaling; iii) Wnt-RAP1 signaling; iv) Wnt-receptor tyrosine kinase-like orphan receptor 2 (ROR2) signaling; v) Wnt-protein kinase A signaling; vi) Wnt-GSK-3-microtubule signaling; vii) Wnt-atypical protein kinase C (PKC) signaling; viii) Wnt-receptor-like tryosine kinase signaling; and ix) Wnt-mammalian target of rapamycin signaling (12). These classifications are not rigid since the pathways overlap and intersect with one another and are evolving. Wnt5a is involved in various cellular functions by activating multiple signaling pathways. However, the role of Wnt5a in cancer metastasis appears to be more complex. Nevertheless, a previous study (13) indicated that Wnt5a plays a key role in malignant progression, although whether Wnt5a exhibits a tumor metastasis-suppressing effect or a promoting effect remains unclear.

## 3. Metastasis-promoting activity of Wnt5a and the underlying mechanisms

WNT5A has been identified as an oncogene in numerous types of tumors. Kurayoshi *et al* (14) detected the expression of Wnt5a in 237 cases of primary gastric cancer via immunohistochemistry. The results revealed that the expression of Wnt5a was upregulated in 30% (71/237) of patients with gastric cancer, which positively correlated with the T grade (depth of invasion) and N grade (degree of lymph node metastasis). The positive rate of Wnt5a expression in stage III/IV gastric cancer tissues (49.5%) was significantly higher than that in stage I/II gastric cancers (13.8%). These results indicated that the upregulation of Wnt5a may be associated with cancer infiltration and lymph node metastasis. In addition, the positivity of Wnt5a expression correlated with advanced stages and poor prognosis of gastric cancer. The authors further demonstrated that Wnt5a had the ability to stimulate cell migration and invasion in gastric cancer cells. Cell migration, membrane ruffling and turnover of paxillin were suppressed in WNT5A knockdown cells. Wnt5a activated focal adhesion kinase and small GTP-binding protein Rac, both of which are known to play a role in cell migration. Laminin γ is also required for liver metastasis of gastric cancer cells *in vivo*. A recent study showed that an anti-Wnt5a polyclonal antibody (pAb5a-5) inhibited the activation of Rac1 and the expression of laminin γ2, which decreased Wnt5a-dependent internalization of the receptor, thereby suppressing the metastasis of gastric cancer cells (15). In addition, Wnt5a promoted the migration of gastric cancer cells via the phosphatidylinositol 3-kinase/protein kinase B/GSK-3β/RhoA signaling pathway (16). Therefore, the expression of Wnt5a in gastric cancer cells may be critical for the migration and invasion of cancer cells from primary regions. Thus, the expression of Wnt5a correlates with the aggressiveness of the gastric cancer.

Gene expression profiling has indicated that WNT5A may be a marker of aggressive in melanomas (17). Immunohistochemical analyses revealed that Wnt5a staining was negative in the majority of benign tumors and was heterogeneously distributed among primary melanomas, while the majority of metastatic tumors were stained strongly positive for Wnt5a. Wnt5a overexpression correlates significantly with the survival and the development of metastases in melanomas. Da Forno *et al* (18) detected the expression levels of Wnt5a and p16ink4a in 59 cases of primary melanoma and their matched metastatic tissues. With tumor progression, the expression of Wnt5a in the cytoplasm gradually increased, however, the expression of p16ink4a was reduced, indicating that overexpression of Wnt5a in the cytoplasm was positively correlated with the progress of the melanoma, as well as poor prognosis. Recently, Grossmann *et al* (19) identified a novel mechanism of action for Wnt5a, by which Wnt5a binds to the cellular Fzd 4-LRP6 receptor complex and activates ADP-ribosylation factor 6, thus, inducing the disruption of the N-cadherin and β-catenin complex and resulting in the enhancement of melanoma invasion and metastasis.

The homeodomain transcription factor, CUTL1, functions as a target of transforming growth factor-β and an important mediator via its effects on cell migration and invasiveness (20). In pancreatic carcinomas, Wnt5a is upregulated by CUTL1 on an mRNA and protein level. Increased activity of a luciferase construct containing the putative Wnt5a promoter upon CUTL1 overexpression indicates that CUTL1 transcriptionally activates Wnt5a. In addition, Wnt5a is a crucial downstream mediator of tumor cell migration, invasion and proliferation induced by CUTL1. Wnt5a is upregulated early during pancreatic carcinogenesis in pancreatic intraepithelial neoplasias lesions and in invasive pancreatic adenocarcinomas, as compared with normal pancreatic tissues. The effects of Wnt5a are also accompanied with epithelial-mesenchymal transition (EMT). These results indicate that WNT5A is an important target of CUTL1 and functions as a novel mediator of invasiveness and tumor progression in pancreatic cancer (21).

Certain studies (22–23) have clarified the association between Wnt5a and tumor metastasis from the aspects of cell polarization and directional movements, since a shared feature of a number of noncanonical Wnt pathways is the targeting of the cytoskeleton, with important implications for cancer metastasis. Witze *et al* (24) investigated the mechanism of how Wnt5a polarizes the cytoskeleton to promote directional motility in cultured melanoma cells. In response to Wnt5a, several proteins, including a melanoma cell adhesion molecule, a Fzd receptor and cytoskeletal components (action microfilaments and a nonmuscle myosin, which are together called actomysin), become polarized in overlapping bands within the trailing edge of the migrating cells. Polarized bands of actomyosin contract to lift the trailing edge, with cell process extensions at the leading edge, and then promote migration toward a chemokine source. Wnt5a functions through the small guanosine triphosphatase (GTPase), RhoB, which controls the movement of intracellular vesicles called endosomes. The authors also observed that an enrichment of multivesicular bodies existed in the polarized cell edge, and polarization required the Rab4 GTPase (known to regulate multivesicular body formation) and dynamin (known to control the formation of endosomes at the cell surface). In conclusion, Wnt5a polarizes cells by promoting the recycling of membrane components to specific surface sites. The concurrent or subsequent recruitment of actomyosin and other factors to these sites defines the trailing edge of the cell, thus, the direction of migration. However, the mechanisms underlying the coupling of membrane polarization to the cytoplasm and the targeting of various proteins to the same sites remain unclear. In addition, it remains unknown how Wnt5a signaling is interfaced with the graded chemokines to ensure the polarization occurs at the cell surface distal to the chemokine source remains unknown (25).

**Figure 1:**
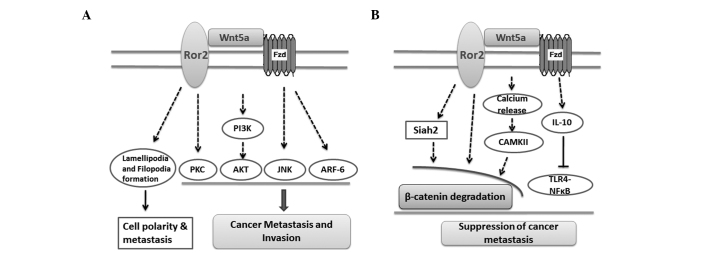
Roles of Wnt5a in cancer metastasis- (A) promoting and (B) suppressive processes. Fzd, frizzled; PI3K, phosphatidylinositol 3-kinase; PKC, protein kinase C; AKT, protein kinase B; JNK, c-Jun N-terminal kinase; ARF-6, ADP-ribosylation factor 6; IL-10, interleukin-10; CAMKII, Ca^2+^/calmodulin-dependent protein kinase II; TLR4, Toll-like receptor 4; NFκB, nuclear factor κB; ROR2, receptor tyrosine kinase-like orphan receptor 2.

An early study of gene expression profiling found that Wnt5a/PKC signaling was associated with aggressive melanoma behavior (17). The Weeraratna group (26) used microarray analysis to demonstrate that Wnt5a directly promoted cell invasion and motility via the inhibition of metastasis-associated gene expression (KISS and CD44) and the stimulation of EMT (a key step in metastasis) in a PKC-dependent manner. Nomachi *et al* (27) found that ROR2, an orphan receptor belonging to the ROR family of receptor tyrosine kinases, played critical roles in Wnt5a-induced cell migration by regulating the formation of lamellipodia and the reorientation of the microtubule-organizing center (MTOC). ROR2 possesses an extracellular cysteine-rich domain that resembles the Wnt-binding sites of the Fzd proteins, thus, it is possible that ROR2 interacts with members of the Wnt family (28). RORs play critical roles in enhancing cell invasion and migration in multiple cancer types (29). Wnt5a stimulation induces the activation of c-Jun N-terminal kinase (JNK) at the wound edge in a ROR2-dependent manner, and inhibiting JNK activity abrogates Wnt5a-induced lamellipodia formation and MTOC reorientation. Additionally, the association of ROR2 with the actin-binding protein filamin A is required for Wnt5a-induced JNK activation and polarized cell migration. Thus, JNK may play roles in cell migration and invasion by phosphorylating the focal adhesion-associated protein, paxillin (30). Further study revealed that Wnt5a-induced JNK activation and MTOC reorientation can be suppressed by inhibiting PKC (31). Therefore, Wnt5a exhibits metastasis-promoting activities in several types of cancer through different signaling pathways (Fig. 1). These novel findings demonstrate the diversity of the noncanonical signaling pathways, as well as further the understanding of tumor metastasis.

## 4. Metastasis-suppressive activity of Wnt5a and the underlying mechanisms

In addition to the tumor metastasis promoting roles of Wnt5a, there is evidence indicating that increased Wnt5a expression is important for the suppression of metastasis in a variety of cancer types, including colon, hematopoietic, breast and thyroid cancers, as well as esophageal squamous cell carcinoma. In the canonical Wnt signaling pathway, accumulation of β-catenin activates the transcription of specific genes in tumor progression (32). Wnt5a is able to antagonize the canonical Wnt signaling pathway, promote the degradation of β-catenin and inhibit tumor invasion and metastasis (33). During esophageal squamous cell carcinoma pathogenesis, Wnt5a is frequently silenced by promoter methylation and antagonizes the Wnt/β-catenin pathway to exhibit tumor suppressor properties (34). Kremenevskaja *et al* (35) reported that Wnt5a increased the release of intracellular calcium ions via the Ca^2+^/calmodulin-dependent protein kinase II (CAMKII) signaling pathway to promote β-catenin phosphorylation and accelerate degradation, thereby inhibiting the translocation and expression of oncogenes, including c-myc. In colonic epithelial cancer cells with truncated APC, activation of the calcium-sensing receptor (CaSR) by ionized Ca^2+^ ions resulted in the upregulation of WNT5A transcripts and the increase in Wnt5a protein secretion. The secreted Wnt5a protein then activated the orphan tyrosine kinase, ROR2, or an uncharacterized Fzd receptor in an autocrine manner to increase the mRNA and protein expression levels of the E-ubiquitin ligase, SIAH2. SIAH2 then mediated growth of β-catenin degradation and reduced β-catenin signaling. When full-length APC was overexpressed in these cells, Ca^2+^ stimulation of the CaSR did not upregulate Wnt5a or increase SIAH2 protein expression (36). These observations indicated that Wnt5a secretion stimulated by CaSR activation may inhibit Wnt signaling in an autocrine manner. Specific studies showed that the inhibitory effect of Wnt5a was not dependent on GSK-3β (37). Jöhnsson and Andersson (38) transfected non-WNT5A expressing MCF-7 breast cancer cells with a mammalian vector carrying WNT5A cDNA. The production of Wnt5a protein in MCF-7 cells was accompanied by an increased ability of discoidin domain receptor 1 (DDR1) phosphorylation and by the improvement of the cancer cell phenotype. DDR1 not only functions as an adhesion receptor, but also increases the number of collagen matrix cells and connections, which can trigger cell signaling and cytoskeleton rearrangement. The study also demonstrated that Wnt5a protein participated in the regulation of adhesion and migration via collagen, and was necessary for the collagen-induced activation of DDR1 in mammary epithelial cells. Dejmek *et al* (39) observed loss or downregulation of Wnt5a expression in 50% of Dukes’ B tumors. Wnt5a negativity was a strong predictor of an adverse outcome, with a relative mortality risk of 3.007 (95% confidence interval, 1.336–6.769; P=0.008) after five years in Wnt5a-negative patients, while the median survival time following diagnosis was 109.1 months for patients with Wnt5a-positive primary tumors. The expression of Wnt5a was decreased in the highly metastatic SW620 human colon cancer cell line as compared with the nonmetastatic SW480 human colon cancer cell line. Furthermore, silencing WNT5A in the highly invasive human colon cancer cell line may contribute to transcriptional regulation by histone modification (40). Additionally, the expression of recombinant Wnt5a significantly reduced the migratory capacity of SW480 cells, the cells that do not express Wnt5a protein possess the ability of high-invasive and metastasis as well as poorly differentiated. However, equivalent treatment did not significantly alter the migration in the Wnt5a-expressing Caco-2 colon cancer cell line. Since inactivating the APC mutation in SW480 cells mimics constant activation of canonical Wnt signaling, and the expression of Wnt5a protein is concurrently absent in SW480 cells, it was hypothesized that there may be a certain degree of antagonism between noncanonical Wnt5a expression and canonical Wnt signaling. These observations indicate that the expression of Wnt5a in primary Dukes’ B colon cancer tissue constitutes a good prognostic marker for longer survival. Ying *et al* (41) also reported the tumor-suppressive activity of Wnt5a. The promoter region of WNT5A is a typical CpG island, which is susceptible to deactivation by methylation in hematological malignancies (34).

By analyzing the epigenetic modification of the WNT5A gene in colon cells from normal colon tissues, colon cancer tissues and colorectal cancer (CRC) cell lines, Ying *et al* found that WNT5A is silenced in the majority of CRC cell lines due to promoter methylation (41). However, Wnt5a is expressed in the majority of normal tissues, including the colon, and is unmethylated in normal colon epithelial cells. WNT5A expression was reactivated by pharmacological or genetic demethylation, indicating that methylation directly mediates the silencing. Similarly, WNT5A methylation was frequently detected in CRC tumors (14/29, 48%), but only occasionally in paired normal colon tissues (2/15, 13%; P=0.025). Ectopic expression of Wnt5a, but not its nonfunctional short-isoform with the Wnt domain deleted, in CRC cells resulted in the substantial inhibition of tumor cell clonogenicity, which is associated with downregulated intracellular β-catenin protein levels and a concomitant decrease in β-catenin activity. Consistent with the results from previous studies, transfection of WNT5A in the FTC-133 thyroid tumor cell line was able to reduce proliferation, migration, invasiveness and clonogenicity in the cells (35). These effects of Wnt5a overexpression are mediated through an increase in intracellular Ca^2+^ release and β-catenin phosphorylation via CaMKII pathways, which result in membranous β-catenin translocation and c-myc oncogene suppression (42). Therefore, Wnt5a serves as an antagonist to the canonical Wnt signaling pathway, exhibiting tumor suppressor activity in differentiated thyroid carcinomas.

Analysis of human primary leukemia cases identified that a deletion of the WNT5A gene and/or loss of Wnt5a expression was present in the majority of the patient samples. Wnt5a hemizygous mice developed myeloid leukemia and B-cell lymphoma, that are common in clonal origin, and exhibited a loss of Wnt5a function in tumor tissues. Wnt5a signaling through the noncanonical Wnt/Ca^2+^ pathway suppresses cyclin D1 expression and negatively regulates B cell proliferation in a cell-autonomous manner (43). Ying *et al* (44) revealed hypermethylation in the promoter of WNT5A in six acute lymphoblastic cell lines (TOM-1, NALM-20, MY, LOUCY, JURKAT and TANOUE), as well as in 43% (132/307) of the acute lymphoblastic leukemia cases. Hypermethylation was found to be associated with the upregulation of cylin D1. As aforementioned, Wnt5a functions as an effector of tumor metastasis suppressive actions via various pathways or by epigenetic changes in its promoter (Fig. 1).

## 5. Contradictory effects of Wnt5a in breast cancer metastasis

Contradictory effects of Wnt5a have been demonstrated in breast cancer. There is increasing evidence that Wnt5a is a tumor suppressor in breast cancer. Jönsson *et al* (45) identified the loss of Wnt5a expression in 59 out of 96 cases of invasive ductal breast cancer, which was associated with high-histological grade, high-mitotic index and negative expression of the estrogen and progesterone receptors. In addition, 78% of the 32 relapse cases exhibited a lack of Wnt5a expression, whereas only 35% of the 51 non-recurrent cases lost Wnt5a expression, and that recurrence-free survival was obviously shorter in the Wnt5a-negative group. Based on the results indicating the inhibition metastasis ability of Wnt5a, FOXY-5, a Wnt5a peptide agonist was developed and tested. Recombinant Wnt5a and FOXY-5 suppressed cell migration and invasion *in vitro*. While *in vivo*, the metastasis of breast cancer cells that had been injected into the mice fat pad was also inhibited by intraperitoneal injection of FOXY-5 into the mice (46,47). The suppression phenotype induced by Wnt5a is associated with the induction of interleukin-10 and the inhibition of the Toll-like receptor 4-nuclear factor κB signaling pathway (48). However, conflicting results remain. Lejeune *et al* (49) reported that the mRNA expression level of WNT5A was upregulated by ten and four times in benign and invasive breast cancer, respectively, as compared with normal breast tissues. In addition, the tumor tissue was Wnt5a negative in 10 cases of the 17 ductal invasive breast cancer patients. There is increasing evidence indicating that interactions between tumor cells and the stromal compartment are critical for malignant progression. Wnt5a appears to promote malignant progression in breast cancer in a microenvironment-dependent manner. Overexpression of Wnt5a has been demonstrated not only in tumor cells, but also in the tumor stroma, particularly tumor-associated macrophages. Wnt5a is detectable in macrophages from metastatic lymph nodes, and Wnt5a-positive macrophages are detected specifically at the invasive front of lymph node metastases. *In vitro* experiments that co-cultured MCF-7 breast cancer cells and macrophages resulted in the upregulation of Wnt5a in the macrophages. In the context of breast cancer, Wnt5a induces invasiveness of cancer cells and the production of matrix metalloproteinase-7 and tumor necrosis factor-α in macrophages. Thus, Wnt5a is essential for macrophage-induced invasiveness in breast cancer. The function of Wnt5a as a suppressor or as a promoter of malignant progression is determined not only by specific intracellular signaling pathways, but also by intercellular interactions among various cell types (50,51).

## 6. Conclusion

Although the results discussed appear conflicting, it is conceivable that the roles of Wnt5a in cancer metastasis. Wnt5a plays a variety of roles in different types of tumors, depending on the binding receptors, downstream effectors, exogenous inhibitors, tumor microenvironments and the extracellular matrix (39), particularly the cell/tissue-tropic contexts (52). To further clarify the role of Wnt5a in tumor metastasis, a number of questions require investigation. It is necessary to reveal the detailed regulatory mechanisms of Wnt5a on cross-talking between the canonical and noncanonical pathways. Further investigation of Wnt5a and the noncanonical Wnt signaling pathways may contribute to revealing the occurrence, development and metastasis of tumors, which may provide novel targets for the diagnosis and treatment of cancer.
